# Therapeutic Potential of Palmitoylethanolamide in Gastrointestinal Disorders

**DOI:** 10.3390/antiox13050600

**Published:** 2024-05-14

**Authors:** Marija Branković, Tijana Gmizić, Marija Dukić, Marija Zdravković, Branislava Daskalović, Davor Mrda, Novica Nikolić, Milica Brajković, Milan Gojgić, Jovana Lalatović, Đorđe Kralj, Ivana Pantić, Marko Vojnović, Tamara Milovanović, Siniša Đurašević, Zoran Todorović

**Affiliations:** 1 University Hospital Medical Center Bežanijska Kosa, 11000 Belgrade, Serbia; gmizic.tijana@bkosa.edu.rs (T.G.); dukic.marija@bkosa.edu.rs (M.D.); zdravkovic.marija@bkosa.edu.rs (M.Z.); mrda.davor@bkosa.edu.rs (D.M.); nikolic.novica@bkosa.edu.rs (N.N.); brajkovic.milica@bkosa.edu.rs (M.B.); lalatovic.jovana@bkosa.edu.rs (J.L.); zoran.todorovic@med.bg.ac.rs (Z.T.); 2 Faculty of Medicine, University of Belgrade, 11000 Belgrade, Serbia; tamara.alempijevic@med.bg.ac.rs; 3 Goodwill Pharma d.o.o, 24000 Subotica, Serbia; branislava.daskalovic@goodwillpharma.rs; 4 University Hospital Medical Center Zvezdara, 11000 Belgrade, Serbia; drkraljdjordje@gmail.com; 5 Clinic of Gastroenterology and Hepatology, University Clinical Center of Serbia, 11000 Belgrade, Serbia; ilic.ivana04@gmail.com (I.P.); marko.vojna@gmail.com (M.V.); 6 Department for Comparative Physiology and Ecophysiology, Institute for Physiology and Biochemistry Ivan Đaja, Faculty of Biology, University of Belgrade, 11000 Belgrade, Serbia; sine@bio.bg.ac.rs

**Keywords:** palmitoylethanolamide, gastrointestinal tract, anti-inflammatory, antioxidants, IBS, IBD

## Abstract

Palmitoylethanolamide (PEA) is an endocannabinoid-like bioactive lipid mediator belonging to the family of N-acylethanolamines, most abundantly found in peanuts and egg yolk. When the gastrointestinal (GI) effects of PEA are discussed, it must be pointed out that it affects intestinal motility but also modulates gut microbiota. This is due to anti-inflammatory, antioxidant, analgesic, antimicrobial, and immunomodulatory features. Additionally, PEA has shown beneficial effects in several GI diseases, particularly irritable bowel syndrome and inflammatory bowel diseases, as various studies have shown, and it is important to emphasize its relative lack of toxicity, even at high dosages. Unfortunately, there is not enough endogenous PEA to treat disturbed gut homeostasis, even though it is produced in the GI tract in response to inflammatory stimuli, so exogenous intake is mandatory to achieve homeostasis. Intake of PEA could be through animal and/or vegetable food, but bearing in mind that a high dosage is needed to achieve a therapeutic effect, it must be compensated through dietary supplements. There are still open questions pending to be answered, so further studies investigating PEA’s effects and mechanisms of action, especially in humans, are crucial to implementing PEA in everyday clinical practice.

## 1. Introduction

The change in nutrition, with regard to high intakes of ultra-processed foods nowadays, led to various chronic diseases, and decreased physical activity just worsened. The consequences are dysnutrition, chronic inflammation, dysbiosis, and immunosenescence, which can further cause metabolic disease, cardiovascular disease, cancer, chronic pain, gastrointestinal disorders, neurodegenerative diseases, and other diseases [1].

Gut health depends on intestinal homeostasis, which further depends on interactions between the intestinal microbiota and the host immune system [2]. As already mentioned, an inadequate diet and the use of antibiotics can lead to dysbiosis, leaky gut, and eventually inflammation [3].

A healthy gut absorbs nutrients without harmful material, such as bacteria and lipopolysaccharide. The intestinal barrier is responsible for the previously mentioned selective absorption of particles into the enteric circulation while preventing bacterial translocation [4,5], but in cases of inflammation, this barrier becomes compromised, leading to leaky gut [6]. For example, inflammation is present in diverticulitis, infective colitis, appendicitis, and inflammatory bowel disease (IBD) [7]. All these conditions are common, and they overwhelm health care systems all over the globe. Unfortunately, there is no available treatment for these permeability changes, but there are several supplements with anti-inflammatory and antioxidant features, so their role is of high clinical importance.

**Figure 1:**
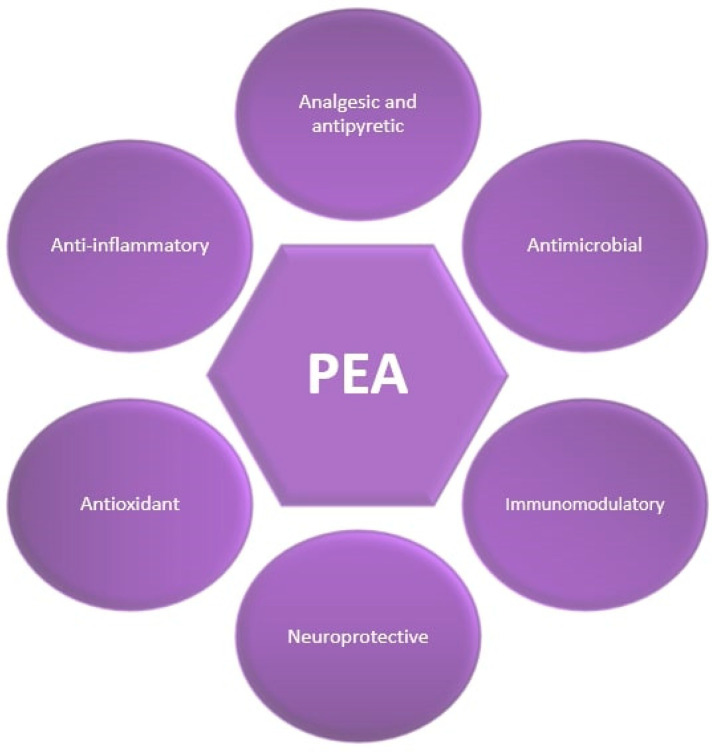
Beneficial effects of Palmitoylethanolamide (PEA).

Palmitoylethanolamide (PEA) is an endocannabinoid-like bioactive lipid mediator belonging to the family of N-acylethanolamines (NAEs) [1,8], most abundantly found in peanuts and egg yolk [9]. PEA has various significant effects, such as anti-inflammatory, antioxidant, analgesic, antimicrobial, antipyretic, immunomodulatory, and neuroprotective activities (Figure 1) [1,10,11].

PEA manifests these numerous effects through multiple pathways at different sites. First of all, it targets the nuclear receptor peroxisome proliferator-activated alpha (PPAR-α), G protein-coupled receptor 55 (GPR55). and G protein-coupled receptor 119 (GPR119) [1]. Additionally, cannabinoid receptors 1 and 2 (CB_1_ and CB_2_) are indirectly activated as a result of inhibition of the degradation of the endocannabinoid anandamide (AEA), also known as the “entourage effect” [1]. Moreover, PEA affects the transient receptor potential vanilloid receptor 1 (TRPV1) channels, leading to analgesic effects. This is thanks to the entourage effect, through PPAR-α activation, and because PEA is potentially acting as an allosteric modulator [12]. What is also important to emphasize is that PEA is able to inhibit mast cell activation [13].

Initially, some studies have shown that a component of egg yolk could have a good effect on rheumatoid arthritis, so this led to a study that identified PEA and showed that it was efficacious in a local passive joint anaphylaxis assay in the guinea pig [14,15,16]. These results motivated other researchers to conduct more clinical studies, and it was concluded that PEA may benefit in the treatment of various conditions, primarily those including pain [12].

Today, PEA has various indications such as muscle recovery, allergic reactions, influenza, common cold, pain, psychiatric, and neurodegenerative conditions [1], and there will certainly be more in the near future. There is not enough endogenous PEA to treat disturbed homeostasis so the exogenous intake would compensate endogenous levels to benefit the homeostasis.

The aim of this literature review is to show how PEA’s anti-inflammatory, antioxidant, analgesic, antimicrobial, and immunomodulatory features can benefit the gastrointestinal tract. The Medline and PubMed databases were searched. Articles were searched according to the keywords “Palmitoylethanolamide”, “PEA”, “antioxidant”, “gastrointestinal tract”, “anti-inflammatory”, “irritable bowel syndrome”, “IBS”, “inflammatory bowel diseases”, “ulcerative colitis”, and “Crohn’s disease”. Both review articles and original articles were considered. The year of publication filter was not used (Table 1).

## 2. Therapeutic Effects of Palmitoylethanolamide (PEA)

### 2.1. The Endocannabinoid System

The endocannabinoid system (ECS) is essential for the proper functioning of the human body. Its importance is highlighted by its extensive distribution throughout the body and its involvement in several physiological processes, including pain, hunger, mood, and immune system control [35]. The ECS is made up of G protein-coupled cannabinoid receptors CB_1_ and CB_2_ [36], their endogenous ligands such as classical endocannabinoids (e.g., anandamide or N-arachidonoylethanolamine and 2-arachidonoylglycerol) and endocannabinoid-like compounds (e.g., PEA, oleoylethanolamide—OEA, and stearoylethanolamide—SEA) [37], and the metabolic enzymes responsible for endocannabinoid synthesis and degradation [38]. The orphan G-protein-coupled receptor GPR55 and two more receptor classes have been introduced to this system as cannabinoid ligand targets: TRPV1 [39] and PPAR nuclear receptors [40].

CB_1_ receptors are found throughout the central nervous system (CNS), including the brain and spinal cord. They are particularly numerous in brain regions linked with memory, cognition, motor function, pain perception, and food management. CB_1_ receptors are prevalent in several brain areas, including the hippocampus, basal ganglia, and cerebral cortex [41]. CB_1_ receptors are also located in peripheral tissues such as adipose tissue, the liver, and skeletal muscles. CB_1_ receptors in peripheral tissues regulate metabolic processes such as lipid metabolism and energy balance. Furthermore, CB_1_ receptors are found in the gastrointestinal system, where they regulate digestion processes and hunger regulation. The location of CB_1_ receptors in the CNS and peripheral tissues explains their role in a variety of processes, including mood regulation, pain modulation, and hunger management [38]. CB_2_ receptors are widely expressed in immune cells such as macrophages, T cells, and B cells, and in the immune system’s peripheral organs, including the spleen, tonsils, and bone marrow. Their presence in these tissues suggests that they have a function in immune response regulation and inflammation. CB_2_ receptors regulate the immune system’s response to injury, infection, and inflammation. Activating CB_2_ receptors may have anti-inflammatory and immunomodulatory properties [42].

TRPV1 has a critical function in pain, nociception, and heat perception [43]. It was initially discovered in primary afferent nociceptors in the dorsal root ganglia, trigeminal ganglia, and vagal ganglia [44]. TRPV1 was later discovered in several areas of the central nervous system, including dopaminergic neurons in the substantia nigra, the hippocampus, hypothalamus, cortex, cerebellum, dentate gyrus, and nucleus accumbens. It is also found in non-neuronal cells such as epidermal keratinocytes, urothelium, hepatocytes, polymorphonuclear granulocytes, pancreatic B cells, endothelial cells, mononuclear cells, smooth muscle cells, mesenteric arteries, pre-adipocytes, and adipose tissue [45]. Xenobiotics, such as capsaicin and piperine, activate TRPV1, as can high temperatures and low extracellular pH. Furthermore, it is worth noting that TRPV1 is heavily regulated and sensitized in the presence of inflammatory situations. This increased sensitivity to stimuli aids in the development and maintenance of intestinal inflammatory processes [46].

Peroxisome proliferator-activated receptors (PPARs) are transcription factors that activate when attached to ligands. They play an important role in regulating the expression of genes required for cellular development and a variety of metabolic activities [47]. Following contact with their respective ligands, a companion receptor, the retinoid X receptor, forms a complex with a varied collection of coactivators. The receptors are transported to the nucleus, where they regulate gene expression. The PPAR family consists of three isoforms: α, δ (also known as β), and γ. Their binding recruits’ other regulatory proteins that modulate transactivation [48]. PPARs play a critical role in inflammation [49], modulating the inflammatory response via a variety of mechanisms, including the suppression of pro-inflammatory substances (e.g., leukotrienes and interleukins) [50]. As a result, it is well established that animals lacking PPARs have a prolonged duration of inflammation [50]. PPARα is located in metabolically active organs like the liver and muscle, where it regulates fatty acid catabolism and inflammation [49]. PPARα plays a direct role in inflammation by inhibiting critical inflammatory transcription factors. It directly affects the pro-inflammatory signaling cascade by targeting NF-κB, AP-1, and STATs. Furthermore, this receptor can catalyze the breakdown of lipid mediators such as leukotriene B4 [51]. PPARα activates the transcription of anti-inflammatory proteins, including IκB-α, by binding to DNA regions [52].

G protein-coupled receptors (GPCRs) are proteins with an intermediate section with seven transmembrane domains. When a ligand interacts with GPCRs, it causes a conformational shift in the transmembrane region, activating the C-terminal and thus the G-protein linked with the GPCR. Depending on the ligand, active G-proteins begin a range of intracellular processes [53]. GPR55 has been found in a variety of tissues, including the brain, particularly in areas associated with memory, learning, and motor activities, as well as the ileum, testicles, amygdala, breast, omental adipose tissue, and several endothelial cell lineages [54]. Its homologs have been found in rats and mice in different brain regions (prefrontal cortex, hippocampus, thalamic nuclei, brainstem, and mesencephalic regions), as well as in peripheral tissues such as the spleen, adrenal glands, and jejunum [55].

### 2.2. Palmitoylethanolamide’s (PEA’s) Pharmacological Profile

PEA is composed of a fatty acid (N-Acyl) linked to ethanolamine. PEA synthesis begins with the transfer of a fatty acid from membrane-bound phospholipids to phosphatidylethanolamine (PE), which is mediated by a calcium ion and cyclic AMP-regulated N-acyltransferase [56], yielding the precursor N-acyl-phosphatidylethanolamine (NAPE). The second stage involves cleaving membrane-bound NAPE to liberate free PEA using N-acyl-phosphatidylethanolamine-selective phospholipase D (NAPE-PLD) [57]. PEA is broken down into palmitic acid and ethanolamine by two different hydrolytic enzymes: fatty acid amide hydrolase (FAAH) and N-acyl-ethanolamine-hydrolyzing acid amidase (NAAH) [58]. Enzymatic activities vary by tissue: FAAH expression may be higher in the brain and liver, while NAAH is found in the colon and macrophages [59].

PEA is known to have a lipophilic nature, being essentially insoluble in water and having a log P larger than 5 [59]. As a result, oral absorption of PEA is highly complex, limited by the dissolving rate, and inversely related to particle size [60]. There has also been little research regarding PEA bioavailability, and there is no clear understanding of how it varies by individual. A study on the oral treatment of male Wistar rats with 100 mg/kg of PEA discovered that the bioavailability of PEA was low, around 25%, but the volume of distribution exceeded the plasma volume, indicating that most of the PEA would be outside the blood after oral administration [61]. PEA levels are highest in peripheral organs such as the adrenal glands, diaphragm, spleen, kidney, testis, lung, liver, and heart, with lower levels in the brain and plasma [62]. These findings show PEA’s ability to cross the blood-brain barrier, emphasizing its importance in brain function.

### 2.3. Palmitoylethanolamide’s (PEA’s) Mechanisms of Action

PEA does not bind to traditional cannabinoid receptors, although it does indirectly stimulate endocannabinoids. It inhibits the enzyme that catalyzes the degradation of AEA, resulting in greater amounts of AEA in tissues and improved analgesic efficacy [63]. PEA binds to PPAR-α [64], TRPV1 [65], and “CB_2_-like” receptors [66], causing an agonistic action. PEA also acts as a GPR55 agonist and directly activates PPARα [67], and it helps to reduce inflammation by inhibiting mast cell activation and lowering the activity of proinflammatory enzymes such as eNOS, iNOS, and COX [66]. Understanding the multiplicity of targets is the key to PEA, as its therapeutic effects can be attributed to a single mechanism or several primary targets.

#### 2.3.1. Pain Management

PEA’s broad-spectrum analgesic, anti-inflammatory, and neuroprotective properties make it an intriguing drug for pain control [68]. In a systematic review and meta-analysis on the PEA effects on chronic pain, 253 unique articles were identified with a combined sample size of 774 patients [69]. PEA was found to reduce pain scores relative to comparators in a pooled estimate, with a standard mean difference of 1.68 (95% CI 1.05 to 2.31, *p* = 0.00001). In another clinical study, when migraine symptoms began, participants were given either 600 mg of PEA or a placebo. After taking the dose, participants rated their pain on a visual analog scale (VAS) every 30 min for 4 h, or until the migraine faded. If the migraine persisted for more than 2 h after the first treatment, participants were asked to take another dose. PEA supplementation relieved more headaches after 2 and 8 h, had a lower VAS for pain score at 1.5 and 4 h, and significantly reduced the utilization of rescue medication compared with the placebo [70].

Analgesic drug modes of action can be divided into three major mechanisms: peripheral sensitization, central sensitization, and pain modulation. Peripheral sensitization occurs at the level of nociceptors. Due to the peripheral sensitization produced by increased nociceptive input, an inflammatory process is initiated, resulting in the release of proinflammatory cytokines (IL-1, IL-2, IL-6, IL-7, and TNF), chemokines, and neutrophils. As a result, primary hyperalgesia develops [71]. Being a PPAR-α ligand, PEA reduces inflammation by promoting the expression of anti-inflammatory proteins and suppressing proinflammatory cytokines like TNF-α [12].

In contrast, central sensitization occurs at the spinal and supraspinal levels, resulting in pain amplification known as secondary hyperalgesia. This process is characterized by changes in mechanical sensitivity, such as mechanical pain sensitivity, pressure pain threshold, and the presence of allodynia [72,73]. Central sensitization is induced by TRPV1-bearing primary afferent activation [74]. Furthermore, microglia and astrocytes play a significant role in modifying synaptic plasticity, which leads to central sensitization [75]. PEA influences both the TRPV1 and glial pathways, normalizing microglial and glial activation and glial interleukin 10 expression [76]. Ambrosino et al. discovered TRPV1 activation and desensitization in PEA and concluded that it caused more TRPV1 desensitization than capsaicin [77]. This process could help explain why PEA reduces central sensitization.

Conditioned pain modulation (CPM) reflects changes in nociceptive processing and captures endogenous pain modulation [78]. It is well established that modification of the noradrenergic transmission pathway influences endogenous pain inhibition [79]. The cannabinoid system got more attention in this context since it has been shown to affect the descending noradrenergic systems [80]. PEA has been shown to decrease Aδ- and C-fiber activity while inhibiting nociceptive-evoked responses of dorsal horn wide-dynamic-range neurons [81].

#### 2.3.2. Anti-Inflammatory Effects

PEA has been widely researched for its interaction with PPARα. This interaction is a fundamental mechanism for PEA’s anti-inflammatory properties and possible therapeutic advantages in a variety of medical problems. In a mouse model of carrageenan-induced paw edema, intracerebroventricular injection of PEA reduced peripheral inflammation by activating PPARα. This led to a considerable reduction in pro-inflammatory enzymes such as cyclooxygenase-2 (COX-2) and inducible nitric oxide synthase [82]. PEA’s anti-inflammatory effect was also reported in a rat paw edema model when administered orally to the animals [83]. The chemical alleviated paw edema and heat hyperalgesia while decreasing neutrophil and mast cell infiltration and the production of pro-inflammatory and pro-nociceptive cytokines such as TNF-α, IL-6, and IL-1β. Additionally, iNOS and COX-2 expression decreased, while IκB-α and NF-κB p65 were degraded.

PEA has shown neuroprotective effects in animal models of neurodegenerative disorders like Parkinson’s and Alzheimer’s disease. It works through anti-inflammatory processes, modulates pro-, and anti-apoptotic indicators, and protects neurons from injury [84]. In addition to its involvement in inflammation and neuroprotection, PEA has shown promise in treating behavioral symptoms associated with autism spectrum disorder (ASD). In animal models of ASD, PEA therapy reversed aberrant behavioral traits by acting on PPARα and lowering inflammation and pro-inflammatory cytokine production [85]. PEA’s indirect stimulation of cannabinoid receptors, specifically CB_2_, has been connected to its PPARα-mediated actions. This connection is important in the setting of neuroinflammatory diseases, as PEA may influence microglial activation and migration [86]. PEA’s therapeutic potential extends to disorders like retinopathy, where it greatly decreases inflammation, inhibits retinal neovascularization, and suppresses pro-fibrotic alterations and Müller gliosis. PEA’s favorable effects in these circumstances have been linked to increased PPARα expression [86].

#### 2.3.3. Antioxidant Effects

There is a study that investigated the possible role of NAEs against oxidative damage. They evaluated the in vitro effect of different NAEs (arachidonoylethanolamide (AEA), oleoylethanolamide (OEA), and PEA) on plasma lipid peroxidation and on the activity of plasma paraoxonase (PON1), which is a serum esterase mainly located on high-density lipoproteins (HDL) with antioxidant and anti-inflammatory features [11].

Eventually, they demonstrated that the NAEs did have a protective effect against in vitro plasma lipid peroxidation induced by copper and AAPH [2-azo-bis(2-aminidinopropane) dihydrochloride] [11]. As already mentioned above, this effect against oxidative stress can be attributed to PON1. Additionally, previous studies have explained that reactive oxygen species (ROS) inhibit PON1 activity [11], which leads to a decrease in the antioxidant activity of HDL towards LDL oxidation, but fortunately, this can be preserved by dietary antioxidants [11]. Explained in more detail, they have shown that NAEs are able to manage the lower decrease in activity of PON1 in oxidized plasma, acting protectively on paraoxonase against copper or AAPH-triggered lipid peroxidation [11]. Even though, according to previous studies, it was thought that the antioxidant effect was due to the metal chelating properties of NAEs, this study has shown that other mechanisms are probably involved, as it was proven that NAEs also have a protective role against AAPH-induced plasma oxidation [11]. 

It is important to emphasize that the same authors have conducted one more study in which it was shown that PEA has a protective effect on low-density lipoprotein (LDL) against copper-induced lipid peroxidation [21]. It was hypothesized that this effect could be a result of a direct interaction between PEA and LDL, as they have demonstrated that PEA causes alterations to the conformational features of the LDL protein component [21]. In favor of these facts, another study has demonstrated that PEA plays a critical role in reducing oxidative stress, i.e., neurons exposed to tert-butyl hydroperoxide-induced stress have shown a lower increase of markers of lipid peroxidation in the presence of PEA [20].

## 3. Therapeutic Potential of Palmitoylethanolamide (PEA) in Gastrointestinal Disorders

The demonstration of PEA’s anti-inflammatory potential in gastrointestinal disorders was conducted using a radiation-induced intestinal inflammation model [87]. In irradiated intestinal regions, a study observed an improvement in the lesion site, including a reduction in intestinal wall thickness, collagen deposition, and neutrophil influx. Furthermore, it was noted that PEA inhibited the anti-inflammatory signaling pathways IL-6 and IL-10, which regulate cellular immune systems derived from mast cells, while activating the prothrombin pathway. It is noteworthy that mice with compromised immune systems exhibited the opposite effect of PEA: it inhibited immune responses derived from non-mast cells, increased the signaling of IL-10 and IL-6 against inflammation, and decreased the activation of the prothrombin pathway.

Using a mouse model of non-alcoholic steatohepatitis (NASH), the function of PEA in the digestive system was also established [88]. NASH is a chronic liver disease characterized by inflammation, fibrosis, hepatic steatosis, and hepatocellular carcinoma, among other symptoms. Hepatocellular carcinoma is a likely progression of NASH. PEA effectively suppressed the advancement of the disease, increased the levels of PPAR-α mRNA and protein, alleviated oxidative stress, decreased the expression of genes associated with lipid metabolism (e.g., acetyl-CoA carboxylase 1 (ACC1) and CD36 mRNA), and mitigated the effects of inflammatory mediators (e.g., MPO, iNOS, TNF-α, chemokine ligand 5 (CCL5), and monocyte chemoattractant protein-1 (MCP-1), also inhibiting the activation of the NLRP3 inflammasome).

Another study investigated the therapeutic effects of PEA on enteric inflammation and bowel motor dysfunctions in an Alzheimer’s disease (AD) model in senescence-accelerated mouse-prone 8 (SAMP8) mice. Furthermore, the ability of PEA to modulate the activation of EGCs, which play an important role in the pathophysiology of bowel dysfunctions associated with inflammatory diseases, was investigated [89]. PEA treatment in SAMP8 animals improves colonic motor activity, citrate synthase activity, and intestinal epithelial barrier integrity while decreasing Aβ and α-synuclein accumulation, S100-β expression, and enteric IL-1β and circulating LPS levels. PEA therapy in EGCs reduced the release of S100-β, TLR-4, NF-κB p65, and IL-1β in response to LPS and Aβ stimulation.

As already mentioned, PEA has beneficial effects on pain and inflammation relief. It was demonstrated that it is synthesized in the human body during inflammation and tissue damage [90], and it was even found in the intestinal biopsies of patients with coeliac disease [26]. There is a study showing that PEA was also synthesized in the experimental model of coeliac-like disease induced by methotrexate in rats; more precisely, levels of intestinal PEA are highest in mice with atrophy and lowest when mice are in remission [26]. In another study, it was shown that PEA reduced hypermotility in the experimental model of ileitis in mice, but the authors did not conclude if this was due to a direct effect on nerve and muscle activity or to the anti-inflammatory features of this substance [30].

PEA influences intestinal motility through a cannabinoid-independent mechanism, and it has been shown that it inhibits transit through the small intestine in mice. It is presumed that this effect is tonic, as fatty acid amide hydrolase (FAAH) does the pharmacological inhibition of its degradation, which then delays motility, and as a result of only partial antagonism of CB_1_ receptor antagonism [91].

Additionally, in another study, it was demonstrated that PEA has shown protective effects against intestinal damage in mice who were on a long-term high-fat diet (HFD). Most likely, this was due to modulation of the gut microbiota, immunomodulation, and restoring tryptophan-derived metabolites altered by HFD [22]. In cases of fat overnutrition, inflammation is first triggered in the gut, as it is directly exposed to dietary-derived toxins [92]. A leaky gut caused by HFD leads to endotoxemia and then to systemic inflammation, which is, fortunately, most commonly low-grade. The authors of the mentioned study came to the conclusion that oral supplementation with PEA in HFD mice has led to immunomodulation, thus limiting immune cell recruitment and mast cell activation [22]. In other words, lipopolysaccharides (LPS) and fatty acids activate toll-like receptor 4 (TLR4), which is in this case the exponent of the immune response in the gut during HFD [93]. In conclusion, if PEA is administered, it reduces the levels of LPS in HFD mice, and then decreased expression of intestinal TLR4 is recorded [22].

As already mentioned above, the same authors have shown that the administration of PEA modulates the gut microbiota. They have started from previous studies that found that HFD feeding disrupted the equilibrium of the microbiota by increasing the Firmicutes/Bacteroidota ratio [22,94]. More precisely, HFD feeding influenced a decrease in the prevalence of gut barrier-protecting species and an increase in the prevalence of opportunistic pathogens producing LPS [22,95]. So, PEA reduced the Firmicutes/Bacteroidota ratio, increased the levels of potentially butyrate-producing bacteria, and also increased sensitivity to local butyrate production [22,96]. These effects prevent leaky gut and reduce inflammation. Furthermore, PEA increased the relative abundance of *Turicibacter sanguinis*, which is a short-chain fatty acid (SCFA) producer found to have many beneficial effects on obesity and insulin resistance [22,97].

### 3.1. The Role of Palmitoylethanolamide (PEA) in Irritable Bowel Syndrome (IBS)

Irritable bowel syndrome (IBS) is one of the most common functional gastrointestinal disorders, with a huge impact on patients’ quality of life. The most common symptoms are abdominal pain, bloating, abdominal distention, and changes in bowel habits [98]. What is interesting about this syndrome is that altered bowel movements can include diarrhea and/or constipation, which cannot be explained by a structural or biochemical abnormality [98]. IBS affects 5%–10% of the population, regardless of age, and most often relapse and remission alternate [98].

Its etiopathogenesis and pathophysiology are still unknown, but there are several presumptions. As it is a functional disorder, it could be the consequence of dysregulation of the gut-brain axis [99]. Additionally, IBS is most likely a multifactorial disease, meaning its development and phenotype are related to both genetic and epigenetic factors, such as interactions between the environment and host [100]. More precisely, in genetically predisposed patients, most commonly, diet, microbiota, or stress can lead to leaky gut, which then triggers immune responses, making the above-mentioned symptoms of this syndrome [99]. Furthermore, IBS can also be a result of an imbalance in the endocannabinoid system [33]. As a response to different damaging stimuli, endogenous NAE levels change in the gastrointestinal tract to regulate food intake, energy balance, and intestinal function [91]. As PEA down-regulate mast cell activity, thus participating in the control of inflammation and nociception, this further leads to the conclusion that supplementation with this substance might improve IBS symptoms, the abdominal pain at most [33].

In connection with the aforementioned, there is a pilot study that evaluated the efficacy and safety of the dietary compounds PEA and polydatin in patients with IBS [33]. They have shown that treatment with these two substances is effective in treating IBS and reduces abdominal pain [33]. Since the mechanism of action of dietary supplements in IBS is not known, the authors investigated mast cell infiltration/activation and the peripheral endocannabinoid system [33]. This was a good way of thinking because they did demonstrate that patients with IBS had an increased infiltration of mast cells in the mucosa of the large bowel in comparison to the control group [33]. Confirming the hypothesis, there was a change in the endocannabinoid system in patients with IBS compared with controls. More precisely, in IBS patients, anti-inflammatory fatty acid amide oleoylethanolamide was reduced (PEA was also reduced, but not in a statistically significant manner), and the expression of the peripheral CB_2_ receptor was higher [33]. Even though PEA and polydatin have shown effectiveness in IBS, the previously mentioned mechanisms of action were not confirmed, so the authors concluded that it was through some different pathways [33].

Moreover, PEA and polydatin were shown to be effective on the severity of abdominal pain in IBS but not on the frequency. Fortunately, pain severity is thought to be more relevant than pain frequency when the efficacy of IBS treatment is investigated [33,101]. The problem is that the mechanisms of action in pain relief of these compounds seem to be complex, as only mast cells in close proximity to colonic nerves lead to IBS-correlated abdominal pain and not mast cell number/activation alone [102]. This was not the only study that failed to elucidate the exact mechanism of action because IBS is a multifactorial disease, so a single mechanism is not enough to cover its complexity [102,103,104,105].

As already stated, mast cells participate in the development of IBS because they are crucial in gut homoeostasis [106] and may contribute to sensory-motor dysfunction [107]. Additionally, the onset of IBS symptoms is related to mast cell count and/or activation [108], and this can be confirmed as the treatment options for IBS could be mast cell stabilizers and H1 antihistamines [105,109].

On the other hand, pain, inflammation, secretion, motility, and gut microbiota are potentially regulated by endocannabinoids and may modulate the expression of CB_2_ receptors [110]. What is crucial is that all these factors are part of the pathophysiology of IBS. As cannabinoids have an analgesic effect, it can be presumed that there is a deficiency of the endocannabinoid system in conditions with symptoms like pain or discomfort, in this case, in IBS [110,111]. Here, it is important to emphasize that there are different study results on the connection between the endocannabinoid system and subtypes of IBS. In one small study, it was shown that in IBS patients with diarrhea, higher levels of 2-arachidonoyl-glycerol were recorded and lower levels of OEA and PEA were recorded, but increased levels of OEA were recorded in IBS patients with constipation [34]. But in the PEA/polydatin study, there was no difference between the endocannabinoid levels and subtypes of IBS [33].

In conclusion, when the therapeutic effects of PEA and polydatin are in question, several options should be additionally investigated. Are those centrally related? Or secondary to mast cell stabilization? Or to the modulation of the endocannabinoid system [33]?

### 3.2. The Role of Palmitoylethanolamide (PEA) in Inflammatory Bowel Diseases (IBD)

Crohn’s disease (CD) and ulcerative colitis (UC) are known as Inflammatory Bowel Diseases (IBD). They are chronic diseases of the gastrointestinal tract mediated by immunity. Although there are many similarities in clinical course and disease progression between CD and UC, they have different pathologies. In CD, transmural inflammation can affect any part of the gastrointestinal tract, with skip lesions, and most often affects the terminal ileum and the right colon. On the other hand, when it comes to UC, the disease is limited to the large intestine, extending continuously proximal from the rectum, while in some patients with pancolitis, lesions in the terminal ileum can also be verified, which is a phenomenon called “back-wash-ileitis”. The pathogenesis of IBD is still unclear, but current research points to a dysregulation of the immune response to the gut microbiota in patients with genetic predispositions [112]. Moreover, according to the available literature, by 2019, the prevalence of IBD was estimated to be around 4.9 million cases globally [113]. Unfortunately, there is a significant increase in IBD incidence worldwide that can be attributed to changes in dietary habits, or, in other words, dysnutrition [114,115]. So, in addition to dysregulation of the immune response, genetic predispositions, and environmental factors, diet for sure has a role in the development of IBD [114,116,117,118,119].

For now, approved therapy for IBD includes aminosalicylates (but according to the latest recommendations, they are not used in CD), corticosteroids, and advanced therapy, which includes immunosuppressive agents and several biologics (Table 2).

Notwithstanding, many IBD patients show no clinical improvement with the indicated treatment modalities. Consequently, there is a huge need for additional research on alternative drugs and therapeutic targets for the treatment of these diseases, and this is still an unsolved challenge for gastroenterologists and pharmacologists. In correlation with the above and keeping in mind that PEA exerts anti-inflammatory, antioxidant, antimicrobial, and immunomodulatory effects that would be beneficial in IBD patients, the next few paragraphs will cover an overview of previous research on this topic.

An investigation was conducted using mouse models of dextran sodium sulfate-induced colitis, colonic biopsies from UC patients, and primary cultures of mouse and human enteric glial cells (EGCs) to evaluate the effects of PEA alone or in the presence of specific PPAR-α or PPAR-γ antagonists [120]. PEA therapy alleviated all macroscopic symptoms of UC while lowering the expression and release of all proinflammatory markers examined. PEA’s anti-inflammatory actions were mediated by specific targeting of the S100B/TLR4 axis on ECGs, which resulted in downstream suppression of NF-kB-dependent inflammation. PPAR-α but not PPAR-γ antagonists eliminated PEA effects in both mice and humans.

In order to achieve its anti-inflammatory effect in treating intestinal diseases, PEA needs to be administered in high doses, so it is not routinely used in clinical practice [23]. A group of authors wanted to find a way to overcome this problem, so they genetically modified probiotics that would produce anti-inflammatory molecules, such as PEA, that would then act at the surface of the colonic mucosa [23]. They have engineered *Lactobacillus paracasei F19* with the human *N*-acylphosphatidylethanolamine-preferring phospholipase D gene (pNAPE-LP) to selectively release PEA in the gastrointestinal tract under the boost of ultra-low doses of exogenous palmitate, and they have investigated its therapeutic potential in mice with experimental UC [23]. They have proved that pNAPE-LP and palmitate increased intestinal delivery of PEA, which led to a clinical and histological improvement of the damage score, reduced neutrophil infiltration, decreased release of pro-inflammatory cytokines and oxidative stress markers, and a markedly restored leaky gut [23]. In addition, they have shown that these effects are secondary to PPARα receptors’ activation, which is indirect evidence that PEA has a key role in mediating pNAPE-LP effects [23]. This is one of the very important discoveries because it could implement pNAPE-LP and palmitate as a treatment modality for IBD.

Furthermore, there is a study that investigated the effect of PEA in a murine model of colitis induced by intracolonic administration of dinitrobenzenesulfonic acid (DNBS) [60]. The results were satisfying, as they showed that inflammation triggered the production of endogenous PEA in the colon but also that the administration of exogenous PEA did have an anti-inflammatory effect [60]. As aforementioned, the authors confirmed that intestinal levels of PEA change in response to harmful stimuli, as was also demonstrated before in patients with UC [19,60]. More precisely, they have observed a nearly threefold increase in intestinal PEA levels in mice with induced colitis in comparison to the control group [60]. In addition, they have demonstrated beneficial effects of PEA as it reduced the weight loss in mice, as histopathology findings showed reduced colon injury, as there was decreased activity of a neutrophil infiltration marker (pathognomonic for mice colitis), as it partially restored leaky gut, and last, but not least, as it limited the colonic diffusion of antigen Kiel 67, which is a marker of dysplasia in UC [60,121,122]. What is interesting to emphasize is that the way of administering PEA showed different results. More precisely, when PEA was given intraperitoneally, it was significantly more active than when given orally, due to the presence of *N*-acylethanolamine-hydrolyzing acid amidase (NAAA) and other PEA metabolizers in the gastrointestinal tract [60,91,123].

Again, the mechanism of action needs to be discussed. The authors have shown that in this animal model of colitis, the effect of PEA was counteracted by a CB_2_ receptor antagonist, most likely via the “entourage effect” [60]. On the other hand, they have shown that PEA up-regulated colonic CB_1_ mRNA expression, which contributes to its anti-inflammatory effect, but still, administration of the CB_1_ receptor antagonist did not affect it [60]. Furthermore, G protein-coupled receptor 55 (GPR55) has a role in mediating the pharmacological actions of CBs, and it is expressed in the gastrointestinal tract of rodents [60,124]. The same authors have proved the involvement of GPR55 as they administered its antagonist to mice with induced colitis, and the results have shown reduced beneficial effects of PEA [60].

Moreover, PPARα is also an important grummet when discussing the mechanisms of action as it maintains colon mucosa homeostasis. In addition, its agonists are beneficial in induced colitis in mice [25,28], and its antagonists neutralized PEA’s anti-inflammatory effect in mice with experimental colitis and in patients with UC, as proved by colon biopsies [24]. In regard to these facts, the aforementioned authors have shown that the effect of PEA was also implemented via a PPARα receptor, as they administered its antagonist to mice with induced colitis, and it resulted in a decrease in PEA’s effect [60].

Last but not least, in patients with IBD, increased immunoreactivity of TRPV1 was seen in colon biopsies, and its antagonists had a beneficial effect in mice with induced colitis [27,29]. So the same authors mentioned above found that PEA has shown a stronger anti-inflammatory effect in the presence of the TRPV1 antagonist, meaning that TRPV1 negatively modulates the pharmacological activity of PEA, which is a very significant fact [60]. In another study, the same authors demonstrated that a different TRPV1 antagonist increased the anti-prokinetic effect of PEA, but this was shown in a post-inflammatory experimental accelerated gastrointestinal transit in mice [60,125].

In another study, the combination of PEA and polydatin was investigated, as were its antioxidant and anti-inflammatory features [126]. Eventually, they also demonstrated that PEA and polydatin did have a beneficial effect in mice with induced colitis, as proven by histology; more precisely, in treated mice, epithelial disruption was significantly reduced [126]. In addition, in treated mice, edema, infiltration of neutrophils, and ulcer formation were significantly improved, but there was also a decrease in weight loss and a reduction of myeloperoxidase (MPO) activity [126]. 

On the other hand, this combination of supplements, in contrast to DNBS, significantly reduced nuclear factor kappa B (NF-κB) translocation and inhibited the inhibitory subunit of NF-κB alpha (IκBα) degradation in mice with induced colitis [126]. This is important because NF-κB is a mediator of inflammation [127], and IκBα is phosphorylated by IκB kinase in response to, for example, infection, oxidative stress, and inflammation [128]. Furthermore, the NF-κB pathway controls the release of pro-inflammatory cytokines like interleukin-1 beta (IL-1β) and tumor necrosis factor alpha (TNF-α), which are increased in IBD, and the authors of the same study have shown that PEA and polydatin decreased those cytokines [126].

Furthermore, data suggest that IBD can be a consequence of decreased antioxidant activity or ROS overproduction [129]. Even though the authors of this study claim that PEA alone does not have an antioxidant capacity, as already stated in this review, it was demonstrated that the NAEs did have a protective effect against in vitro plasma lipid peroxidation induced by copper and AAPH [11]. Despite this fact, they have shown that the combination of PEA and polydatin neutralized oxidative stress and ROS formation by decreasing inducible nitric oxide synthase (iNOS) expression, poly-ADP ribose polymerase (PARP), and nitrotyrosine levels, but also by increasing the levels of the antioxidative enzyme manganese superoxide dismutase (MnSOD) [126]. In order to prove that statement, the authors had an idea to investigate the antioxidant pathway of silent information regulator 1/nuclear erythroid factor 2-related factor 2 (SIRT1/Nrf2), which is in correlation with the already explained NF-κB signaling [126]. So far, SIRT1 has been shown to play a role in the regulation of oxidative stress and inflammation, while Nrf2 regulates the transcription of antioxidant proteins, translocates to the nucleus, and promotes heme oxygenase (HO) expression after cell injury [126,130,131,132]. Finally, they showed that administration of DNBS resulted in a decrease in SIRT1, which further led to a reduction in Nrf2 activity and eventually decreased the expression of HO-1 [126]. On the other hand, the administration of PEA and polydatin was shown to inhibit DNBS-induced downregulation of SIRT1, Nrf2, and HO-1 [126]. This was also proven with the administration of sulforaphane (SF), since it can also activate the Nrf2 antioxidant pathway [126,133,134,135]. In conclusion, study results show that PEA and polydatin, but also SF treatment, have increased MnSOD expression levels and the antioxidant activities of Nrf2 in mice with DNBS-induced colitis [126]. Bearing in mind that there is a connection between Nrf2 and NF-κB, it is concluded that PEA and polydatin act anti-inflammatoryly via the NF-κB pathway, while antioxidant activity is achieved by modulating the Nrf2 pathway [126,136,137,138]. 

However, additional and advanced research on PEA’s molecular targets is needed in order to fully understand its mechanism of action and beneficial effects. Anyhow, the exogenous intake of PEA at the allowed dose in patients with gastrointestinal diseases would not be of any harm, for sure [139].

## 4. Conclusions

This literature review has covered various studies about endogenous or exogenous PEA’s beneficial effects on the gastrointestinal tract. Indeed, intake of PEA could be through animal and/or vegetable food; it can be taken as a supplement, but it is also produced in the gastrointestinal tract in response to inflammatory stimuli. Unfortunately, there is not enough endogenous PEA to treat disturbed gut homeostasis, so the exogenous intake could be used to achieve homeostasis.

As a supplement, it was shown that it could be used for several gastrointestinal diseases. In IBS, it reduces abdominal pain intensity and frequency, while in IBD, it controls intestinal inflammation. On the other hand, PEA could also influence gut microbial community assortment. What is also important is that it inhibits intestinal motility and reduces large bowel permeability. These effects are due to the anti-inflammatory, antioxidant, analgesic, antimicrobial, immunomodulatory, and other features of PEA.

In addition, the administration of a genetically modified probiotic (pNAPE-LP) that stimulates the production of PEA in situ at the surface of the colonic mucosa under the boost of ultra-low doses of exogenous palmitate resulted in reduced inflammation, decreased release of pro-inflammatory cytokines and oxidative stress markers, and a reduction of increased intestinal permeability in mice with experimental colitis [23]. This gives hope that this mode of PEA utilization could be implemented as another treatment option for IBD.

Ultimately, there are still open questions pending to be answered, so further studies investigating PEA’s effects and modes of action, especially in humans, are crucial in order to implement PEA in everyday clinical practice.

## Figures and Tables

**Table 1:** Summary table of the main studies classified according to the levels of evidence.

Article Type	Year	Authors	Title	Main Findings	Ref. No.
Reviews	2022	A. K. Kiani et al.	Dietary supplements for intestinal inflammation	The PEA treatment was reported to be noticeably effective in decreasing abdominal pain severity in IBS. PEA is a nutritional compound able to decrease the activation of mast cells.	[9]
	2021	A. Heidari et al.	The immune system and autism spectrum disorder: association and therapeutic challenges	PEA, an endocannabinoid molecule, has potential anti-inflammatory effects. Consistently, it improved autistic-like behaviors by affecting intestinal microbial composition in mice.	[17]
	2016	F. Ochoa-Cortes et al.	Enteric Glial Cells: A New Frontier in Neurogastroenterology and Clinical Target for Inflammatory Bowel Diseases	PEA, by interacting with peroxisome proliferator-activated receptor–α expressed by glial cells, can counteract the increased expression of TLR4/S100B proteins, together with p38/p-ERK/pJNK-pathway signaling molecules, NF-κB expression, and NO release, in patients with ulcerative colitis.	[18]
	2005	N. A. Darmani et al.	Involvement of the cannabimimetic compound, N-palmitoyl-ethanolamine, in inflammatory and neuropathic conditions: review of the available pre-clinical data, and first human studies	Colonic PEA levels in biopsies from patients with ulcerative colitis were found to be 1.8-fold higher than those in healthy subjects.	[19]
In vitro studies	2008	Zolese G. et al.	Effect of Acylethanolamides on Lipid Peroxidation and Paraoxonase Activity	N-acylethanolamides protect plasma lipids and PON1 activity against AAPH and/or copper-induced oxidation.	[11]
	2007	Lombardi G. et al.	Oxyhomologation of the Amide Bond Potentiates Neuroprotective Effects of the Endolipid N-PEA	Oxyhomologation of the amide bond potentiates the neuroprotective effects of the endolipid N-PEA. Also proven is the antioxidant effect of PEA.	[20]
	2005	Zolese G. et al.	Increased Plasma Concentrations of Palmitoylethanolamide, an Endogenous Fatty Acid Amide, Affect Oxidative Damage of Human Low-Density Lipoproteins: An in Vitro Study	Indicate both anti-oxidative and slightly pro-oxidative effects of PEA.	[21]
Murine models	2023	Pirozzi C. et al.	Palmitoylethanolamide Counteracts High-Fat Diet-Induced Gut Dysfunction by Reprogramming Microbiota Composition and Affecting Tryptophan Metabolism	PEA leads to a decrease in inflammatory factors in the gut. The administration of ultra micronized PEA reprograms gut microbial community assortment.	[22]
	2021	Esposito G. et al.	Engineered Lactobacillus Paracasei Producing Palmitoylethanolamide (PEA) Prevents Colitis in Mice	They concluded that pNAPE-LP with ultra-low palmitate supply stands as a new method to increase the in situ intestinal delivery of PEA and as a new therapeutic able to control intestinal inflammation in inflammatory bowel disease.	[23]
	2014	Esposito G. et al.	Palmitoylethanolamide Improves Colon Inflammation through an Enteric Glia/Toll like Receptor 4-Dependent PPAR-α Activation	Because of its lack of toxicity, its ability to reduce inflammation, and its selective PPARα action, PEA might be an innovative molecule to broaden pharmacological strategies against UC.	[24]
	2010	Azuma Y-T. et al.	PPARα Contributes to Colonic Protection in Mice with DSS-Induced Colitis	They suggest that PPARα has a role in controlling colonic inflammation and mucosal tissue homeostasis.	[25]
	2007	D’Argenio G. et al.	Overactivity of the Intestinal Endocannabinoid System in Celiac Disease and in Methotrexate-Treated Rats	The levels of anandamide and PEA were significantly elevated (approx. 2 and 1.8-fold, respectively) in active celiac patients, as were those of CB1 receptors. The levels of anandamide, 2-AG, and PEA peaked 3 days after treatment with Methotrexate and returned to basal levels at remission 7 days after treatment.	[26]
	2005	Dömötör A. et al.	Immunohistochemical Distribution of Vanilloid Receptor, Calcitonin-Gene Related Peptide and Substance P in Gastrointestinal Mucosa of Patients with Different Gastrointestinal Disorders	The immunohistochemical distribution of TRPV1, CGRP, and SP differs in gastrointestinal diseases of the upper and lower tract, and the participation of TRPV1, CGRP, and SP differs significantly in these different gastrointestinal diseases.	[27]
	2004	Cuzzocrea S. et al.	Role of Endogenous and Exogenous Ligands for the Peroxisome Proliferators Activated Receptors Alpha (PPAR-Alpha) in the Development of Inflammatory Bowel Disease in Mice	The absence of the PPAR-alpha receptor significantly abolished the protective effect of the PPAR-alpha agonist against DNBS-induced colitis. Endogenous and exogenous PPAR-alpha ligands reduce the degree of colitis caused by DNBS, so PPAR-alpha ligands may be useful in the treatment of IBD.	[28]
	2004	Kimball E. et al.	Vanilloid Receptor 1 Antagonists Attenuate Disease Severity in Dextran Sulphate Sodium-Induced Colitis in Mice	The results suggest that pharmacological modulation of TRPV1 attenuates indices of experimental colitis in mice and that the development of orally active TRPV1 antagonists might have therapeutic potential for the treatment of IBD.	[29]
	2001	Capasso R. et al.	Inhibitory Effect of Palmitoylethanolamide on Gastrointestinal Motility in Mice	It is concluded that PEA inhibits intestinal motility through a peripheral mechanism independent from cannabinoid receptor activation.	[30]
Clinical Trials	2024	G. Di Nardo et al.	Palmitoylethanolamide and polydatin in pediatric irritable bowel syndrome: A multicentric randomized controlled trial	Co-micronized PEA/polydatin (PEA/PD) demonstrated efficacy in pediatric irritable bowel syndrome, significantly increasing complete remission. Subgroup analysis highlighted benefits in the irritable bowel syndrome-diarrhea subtype. Treatment with PEA/PD resulted in a notable reduction in abdominal pain intensity and frequency compared with placebo.	[31]
	2019	D. G. Couch et al.	Palmitoylethanolamide and Cannabidiol Prevent Inflammation-induced Hyperpermeability of the Human Gut In Vitro and In Vivo-A Randomized, Placebo-controlled, Double-blind Controlled Trial	In vitro, PEA decreased the inflammation-induced flux of dextran and prevented an inflammation-induced fall in TRPV1 and an increase in PPARα transcription. In conclusion. PEA reduces permeability in the human colon.	[32]
	2017	Cremon C. et al.	Randomized Clinical Trial: The Analgesic Properties of Dietary Supplementation with Palmitoylethanolamide and Polydatin in Irritable Bowel Syndrome	The marked effect of the dietary supplement PEA/polydatin on abdominal pain in patients with IBS suggests that this is a promising natural approach for pain management in this condition.	[33]
	2013	Fichna J. et al.	Endocannabinoid and Cannabinoid-like Fatty Acid Amide Levels Correlate with Pain-Related Symptoms in Patients with IBS-D and IBS-C: A Pilot Study	Patients with IBS-D had higher levels of 2AG and lower levels of OEA and PEA. In contrast, patients with IBS-C had higher levels of OEA. Multivariate analysis found that lower PEA levels are associated with cramping abdominal pain. FAAH mRNA levels were lower in patients with IBS-C.	[34]

**Table 2:** Approved treatment modalities for inflammatory bowel diseases (IBD).

Aminosalicylates	Corticosteroids	Advanced Therapy
mesalamine	methylprednisolone	infliximab
sulfasalazine	prednisone	adalimumab
olsalazine	hydrocortisone	golimumab
balsalazide	budesonide	vedolizumab
		ustekinumab
		tofacitinib
		upadacitinib

## Data Availability

Not applicable.
